# Serological and Molecular Investigation on *Toxoplasma gondii* Infection in Wild Birds

**DOI:** 10.3390/pathogens8020058

**Published:** 2019-04-29

**Authors:** Simona Nardoni, Guido Rocchigiani, Ilaria Varvaro, Iolanda Altomonte, Renato Ceccherelli, Francesca Mancianti

**Affiliations:** 1 Dipartimento di Scienze Veterinarie, Università degli Studi di Pisa, 56124 Pisa, Italy; guido.rocchigiani.g@gmail.com (G.R.); ilaria5787@virgilio.it (I.V.); altomonte@vet.unipi.it (I.A.); francesca.mancianti@unipi.it (F.M.); 2 Centro Recupero Uccelli Marini e Acquatici-CRUMA, 57121 Livorno, Italy; apusvet.cruma@libero.it

**Keywords:** *Toxoplasma gondii*, birds, IFAT, serology, PCR, zoonosis, One Health

## Abstract

*Toxoplasma gondii* is an obligate apicomplexan zoonotic parasite that infects humans and other animals and is responsible for toxoplasmosis. This parasite causes one of the most common parasitic infections in humans worldwide. Toxoplasmosis meets the requirements for a One Health Disease due to its ability to affect the health of human beings as well as domestic and free ranging animals. Integrating human, domestic animal, and wildlife data could better assess the risk and devise methods of control. A first step of such an approach would be the knowledge of the prevalence of parasitosis in humans and animals in selected areas. Therefore, the aim of the present study was to evaluate the occurrence of *Toxoplasma* infection in 216 free ranging birds belonging to different genera/species by serology and molecular techniques. Twenty-five out of 216 animals (11.6%) were positive to the immunofluorescence antibody test (IFAT) with antibody titers ranging from 1/20 to 1/320, and 19 of them (8.8%) also showed a positive PCR for *Toxoplasma* DNA. The results confirmed the widespread occurrence of *Toxoplasma* infection in wild birds and serological data were corroborated by molecular results in birds that also had low antibody titers. The knowledge of the wide occurrence of the parasite in game and wild birds should enhance the accurate estimation of the risks in handling, managing, and eating these species with regard to domestic carnivores as well as the impact of viscera and offal in the environment.

## 1. Introduction

*Toxoplasma gondii* is an obligate apicomplexan zoonotic parasite that infects humans and other animals and is responsible for toxoplasmosis. The life cycle of this intracellular parasite is very complex and encounters two stages. The sexual stage develops in the intestine of wild and domestic felid hosts, while the asexual phase takes place in all warm-blooded animals including birds and mammals.

Animal infection can take place by the ingestion of small mammals and birds bearing tissue cysts and/or of oocysts shed by the feline final host and sporulated in the environment. Horizontal transmission in humans follows the oral intake of raw or undercooked meat as well as food and water contaminated by sporulated oocysts.

The parasite is widespread throughout the world, and toxoplasmosis represents one of the most common parasitic infections in humans. Although the infection is most typically asymptomatic in immunocompetent subjects, a number of reports about the occurrence of ocular symptoms such as retinochoroiditis and retinitis consequent to acquired toxoplasmosis in humans are present in the literature. Immunocompromised patients are at risk from severe disease following both primary infection and reactivated toxoplasmosis [1]. If primary infection is contracted during pregnancy, intrauterine infection may occur in immunocompetent women, with transmission to the fetus [2,3].

Toxoplasmosis meets the requirements for a One Health Disease due to its ability to affect the health of human beings as well as domestic and free ranging animals. It also impacts on ecosystems and is a threat to those who rely on animal resources [4,5,6]. Recently, the One Health approach to toxoplasmosis has been excellently revised by Aguirre et al. [6], hoping for transdisciplinary collaborations, by monitoring toxoplasmosis and *T. gondii* prevalence. Integrating human, domestic animal, and wildlife data could better assess the risk and devise methods of control. A first step of such an approach would be acquiring the knowledge of the prevalence of parasitosis in human and animals in selected areas.

Toxoplasmosis in wildlife such as many other parasite zoonoses has little clinical impact, however, the spillover from wildlife to human and/or domestic animals should be considered [7].

Avian species are usually resistant to *Toxoplasma* infection, even if pigeons and canaries can be severely affected [8]. Wild birds are important in the *T. gondii* epidemiology, which considered their role as a reservoir for carnivores. Some of them also have migrating behavior and could spread the parasite worldwide.

Data about the occurrence of *Toxoplasma* infection in wild birds in Italy are scant and to the best of our knowledge, there have only been two reports referring to waterfowl and birds of prey, respectively [9,10]. For these reasons, the aim of the present study was to evaluate the occurrence of *Toxoplasma* infection in various free ranging bird species by serology and molecular techniques.

## 2. Results

Twenty five out of 216 animals (11.6%) were positive to the immunofluorescence antibody test (IFAT) with antibody titers ranging from 1/20 to 1/320, and 19 of them (8.8%) also showed a positive PCR for *Toxoplasma* DNA. In detail, DNA was found in the hearts of 11 birds, in the brains of four animals, while the other four subjects had parasite DNA both in the heart and brain.

Detailed results are reported in Table 1.

Twelve hunted waterfowl out of 148 (8.1%) were seropositive and eight of them (5.4%) harbored parasite DNA, while the birds from a rescue center yielded a seroprevalence of 19.1% and 13.2% gave a positive PCR result.

## 3. Discussion

In the present report, an overall seroprevalence of 11.6% and positive PCR of 8.8% were found. Although the selected bird population was very heterogeneous and the number of subjects was low, these preliminary data are of interest. There have only been a few studies reporting on the occurrence of antibodies or DNA in avian species in Europe, except for Spain. To the best of our knowledge, information about the simultaneous presence of the two data is lacking, except for a previous study carried out by us on waterfowl from Tuscany [9]. In this paper, an 8.7% seroprevalence along with a PCR positivity of 2.9% was reported. The data referring to waterfowl in the present study gave a similar seroprevalence, but the occurrence of parasite DNA was more than twice. Moreover, surprisingly, in the present study, the heart showed a greater sensitivity, yielding positive results for parasite DNA in 15 animals, while the brains were positive in only eight subjects.

Serological studies from Europe regarding raptors showed a prevalence of 50% from Portugal [9], from 0% to 79% in France [11,12] and 10.7% from Italy [10]. Other studies have reported seroprevalence values of 80.5% in common ravens [13], and 26.1% in various avian species from Spain [14]. Molecular studies have been carried out on the brains of different avian species in Spain, with an overall PCR positivity of 6% [15].

The results from this preliminary report would fit with data referred by other countries, and four raptors out of 15 (27%) were seropositive. Four out of 18 seagulls (22.3%) scored seropositive, in full agreement with Cabezon et al. [16] who first reported the occurrence of antibodies (21%) versus *T. gondii* in 525 seagull chicks, and with Gamble et al. [17], who reported a large exposure of yellow-legged gull to the parasite by checking 1122 eggs and hypothesized of their involvement in the maintenance and circulation of toxoplasma, suggesting that large gulls could be used as epidemiological sentinels at the human–wildlife interface.

These results confirm the widespread occurrence of toxoplasma infection in wild birds and the serological data were also corroborated by molecular results in waterfowl with low antibody titers (1/20).

One Health has emphasized the need to bridge disciplines linking human health, animal health, and ecosystem health [4]. Human activities can influence the zoonotic transmission of *Toxoplasma* involving wildlife. Hunting, the lack of control of domestic hosts (diet and roaming), dietary human factors, and environmental contamination are the most important risk factors [7]. The One Health triad encompasses the collaborative goals of providing optimal health for people, animals, and the environment by considering interactions between all three systems [7]. Therefore, knowledge of the wide occurrence of the parasite in game and wild birds should enhance an accurate estimation of the risks in handling, managing, and eating these species also with regard to domestic carnivores as well as to the impact of viscera and offal into the environment.

## 4. Material and Methods

The study was carried out on n = 216 birds (Table 2) belonging to different avian species, referred to the Veterinary Parasitology Lab for epidemiological purposes. Part of them (n = 148) were waterfowl from regular hunting activity. The other birds (n = 68) were referred by a rescue center for avian species (CRUMA) and were injured animals, or who had died during hospitalization.

The whole brain and heart [18] as well as intracardiac coagules were collected from all birds, shortly after their delivery at the Lab.

Tissues were kept at −20 °C until processing for the molecular detection of *Toxoplasma* DNA, and serum was employed to evaluate the presence of anti-*Toxoplasma* antibodies by IFAT, using 12-well slides (Toxospot ^®^, BioMérieux, Marcy l’Etoile, France) and an anti-chicken IgG FITC (Sigma-Aldrich s.r.l., Milan, Italy) diluted 1/32. This test was preferred to MAT, considering that most sera were hemolyzed, making it hard to read the results. Therefore, IFAT was employed, as reported by Maksimov et al. [19], with slight modification, starting from a threshold dilution of 1/20.

DNA was extracted from about 200 mg of homogenized tissues to perform PCR analysis for *Toxoplasma* DNA using the DNeasy^®^ Blood & Tissue (Qiagen, Milan, Italy) in accordance with the manufacturer’s instructions. After extraction, DNA was stored at −20 °C until use.

Two pairs of oligonucleotide primers were used to amplify regions of the B1 gene of *T. gondii*: the outer primers 5’-GGAACTGCATCCGTTCATGAG-3’ (sense strand) and 5’-TCTTTAAAGCGTTCGTGGTC-3’ (nonsense strand) and inner primers 5’-TGCATAGGTTGCAGTCACTG-3’ (sense strand) and 5’-GGCGACCAATCTGCGAATACACC-3’ (nonsense strand), provided by Eurofins MGW (M-Medical, Milano, Italy). Nested PCR was performed as described by Jones et al. [20].

## Figures and Tables

**Table 1:** Species, gender, and the results of the serology and PCR on positive birds.

Bird Species	Gender	IFAT	PCR Heart	PCR Brain
*Anas crecca*	male	1/40	negative	positive
*Anas crecca*	female	1/20	positive	negative
*Anas crecca*	female	1/20	negative	negative
*Anas crecca*	female	1/80	positive	negative
*Anas crecca*	female	1/40	positive	negative
*Anas crecca*	female	1/20	negative	negative
*Anas crecca*	female	1/20	negative	negative
*Anas crecca*	female	1/80	negative	positive
*Anas penelope*	female	1/40	positive	negative
*Anas penelope*	female	1/20	positive	positive
*Anas plathyrinchos*	male	1/20	positive	negative
*Anas plathyrinchos*	female	1/40	negative	negative
*Buteo buteo*	male	1/160	negative	negative
*Columba palumbus*	female	1/80	positive	positive
*Falcus tinnunculus*	male	1/160	negative	negative
*Falcus tinnunculus*	male	1/320	positive	positive
*Falcus tinnunculus*	female	1/320	positive	positive
*Gallinago gallinago*	female	1/40	negative	negative
*Larus ridibundus*	male	1/160	positive	negative
*Larus ridibundus*	female	1/160	negative	positive
*Larus ridibundus*	female	1/320	positive	negative
*Larus ridibundus*	male	1/160	positive	negative
*Phasianus colchicus*	male	1/80	positive	negative
*Sturnus vulgaris*	male	1/80	positive	negative
*Vanellus vanellus*	male	1/80	positive	negative

**Table 2:** Number, species and gender of birds studied.

Species	Total Number	Males	Females
*Anas crecca*	73	23	50
*Anas platyrhyncos*	28	15	13
*Anas penelope*	23	10	13
*Anas acuta*	5	0	5
*Anas strepera*	3	0	2
*Aythya ferina*	3	2	1
*Tadorna tadorna*	3	1	2
*Anas querquendula*	1	0	1
*Aythya fuligula*	1	0	1
*Anas clypeata*	8	2	6
*Anser anser*	1	1	1
*Sturnus vulgaris*	2	2	0
*Corvus frugilegus*	2	1	1
*Sylvia melanocephala*	1	1	0
*Garrus glandarius*	1	0	1
*Spinus spinus*	1	1	0
*Parus major*	1	1	0
*Phylloscopus collybita*	1	0	1
*Columba livia*	3	0	3
*Columba palumbus*	8	6	2
*Falco tinnunculus*	6	4	2
*Falcus peregrinus*	1	1	0
*Phasianus colchicus*	3	2	1
*Gallinago gallinago*	6	3	3
*Vanellus vanellus*	2	2	0
*Larus ridibundus*	18	4	14
*Buteo buteo*	4	2	2
*Asio otus*	1	1	0
*Fulica atra*	2	2	0
*Athene noctua*	3	1	2
*Ardea cinerea*	1	1	0
